# Leaf nutrient traits of planted forests demonstrate a heightened sensitivity to environmental changes compared to natural forests

**DOI:** 10.3389/fpls.2024.1372530

**Published:** 2024-03-18

**Authors:** Xing Zhang, Mengyao Yu, Jianxiao Su, Jiali Xu, Xueting Zhang, Jinlong Shang, Jie Gao

**Affiliations:** ^1^ Key Laboratory for the Conservation and Regulation Biology of Species in Special Environments, College of Life Science, Xinjiang Normal University, Urumqi, China; ^2^ Key Laboratory of Earth Surface Processes of Ministry of Education, College of Urban and Environmental Sciences, Peking University, Beijing, China

**Keywords:** leaf nutrient traits, climate change, soil nutrients, forest age, planted forest, natural forest

## Abstract

Leaf nutrient content (nitrogen, phosphorus) and their stoichiometric ratio (N/P) as key functional traits can reflect plant survival strategies and predict ecosystem productivity responses to environmental changes. Previous research on leaf nutrient traits has primarily focused on the species level with limited spatial scale, making it challenging to quantify the variability and influencing factors of forest leaf nutrient traits on a macro scale. This study, based on field surveys and literature collected from 2005 to 2020 on 384 planted forests and 541 natural forests in China, investigates the differences in leaf nutrient traits between forest types (planted forests, natural forests) and their driving factors. Results show that leaf nutrient traits (leaf nitrogen content (LN), leaf phosphorus content (LP), and leaf N/P ratio) of planted forests are significantly higher than those of natural forests (P< 0.05). The impact of climatic and soil factors on the variability of leaf nutrient traits in planted forests is greater than that in natural forests. With increasing forest age, natural forests significantly increase in leaf nitrogen and phosphorus content, with a significant decrease in N/P ratio (P< 0.05). Climatic factors are key environmental factors dominating the spatial variability of leaf nutrient traits. They not only directly affect leaf nutrient traits of planted and natural forest communities but also indirectly through regulation of soil nutrients and stand factors, with their direct effects being more significant than their indirect effects.

## Introduction

Leaf nutrient traits reflect the nutrient adaptation strategies plants adopt in response to climate change (Wang et al., 2022). Leaf nitrogen (N) and phosphorus (P) content, as well as the leaf N/P ratio, significantly influence plant metabolism and growth processes (Wang et al., 2022). They affect forest ecosystem productivity by influencing photosynthetic intensity, thereby enhancing the environmental stability of forest ecosystems (Malhi et al., 2002). Leaf nitrogen and phosphorus contents are usually associated with plant growth rates and nutritional status (Liu et al., 2023), where higher leaf N and P contents indicate faster plant growth rates (Niklas, 2006). The N/P ratio reflects plant demand for nitrogen and phosphorus (Tessier and Raynal, 2003) and plays an important role in investigating plant competition, ecosystem nutrient cycling, and ecosystem stability (Xu et al., 2020). Numerous studies have shown that abiotic factors such as climate change and soil nutrients play a key role in shaping the spatial variability of forest leaf nutrients (Gong et al., 2023). However, studies also indicate that stand factors like forest age play a significant role as well (Nielsen et al., 2019), and it remains unknown whether different forest origins (planted forest, natural forest) affect leaf nutrient functional traits.

Extensive research has found that climatic factors, especially temperature and rainfall, dominate the spatial variability of forest leaf nutrient functional traits (Zhang et al., 2018).Temperature affects leaf nutrient functional traits by influencing plant physiological and metabolic processes (Ding et al., 2020). An increase in temperature can enhance plant growth rates and nutrient demand, leading to a decrease in leaf nitrogen and phosphorus content. Additionally, high temperatures may affect the activity of rhizosphere microorganisms, reducing the availability of nitrogen and phosphorus in the soil, thereby limiting the plant’s nutrient uptake capacity (Trezzi et al., 2021). Furthermore, precipitation affects leaf nutrient traits by influencing soil moisture supply (Tuttle and Salvucci, 2016; Dai et al., 2022). Increased rainfall typically raises the concentration of dissolved nutrients in the soil, facilitating the absorption of nitrogen and phosphorus by plants. However, under prolonged drought conditions, plants may experience water limitations, leading to a decrease in leaf nitrogen and phosphorus content (Seleiman et al., 2021). Additionally, sunlight is a key factor affecting leaf nutrient functional traits (Lafont Rapnouil et al., 2023). Adequate sunlight can increase the rate of photosynthesis in plants, raising the demand for nutrients and prompting the accumulation of more nitrogen and phosphorus in the leaves (De Souza et al., 2022). However, under conditions of excessively strong or weak light, photosynthesis may be inhibited, leading to a reduction in leaf nitrogen and phosphorus content.

Soil, as the direct living environment of plants, also plays a significant role in forest leaf nutrient functional traits (De Long et al., 2019). Soil nitrogen content directly impacts leaf nitrogen content and the N/P ratio, as soil nitrogen availability determines the plant’s ability to absorb nitrogen (Waring et al., 2023). When soil nitrogen content is high, plants can more easily absorb sufficient nitrogen, leading to increased leaf nitrogen content (Waring et al., 2023). Soil phosphorus content is also crucial for plant nutrient absorption and growth (Kirkby and Johnston, 2008). The availability of phosphorus in the soil determines whether plants can obtain an adequate phosphorus supply. When soil phosphorus content is low, plants face phosphorus limitation, leading to decreased leaf phosphorus content and consequently affecting the leaf N/P ratio. Soil pH has complex effects on leaf nutrient functional traits (Cerozi and Fitzsimmons, 2016). Soil pH affects the chemical form and solubility of nutrients; under different pH conditions, the availability of nitrogen and phosphorus in the soil varies. For example, in acidic soils, the release of ions such as aluminum and manganese may inhibit the availability of phosphorus, thus affecting plant nutrient absorption (Wang et al., 2022). Therefore, changes in soil pH may impact leaf nitrogen and phosphorus content as well as the N/P ratio.

Besides climatic and soil nutrient factors, stand factors such as forest age and stand density also significantly affect leaf nutrient functional traits (Zhang et al., 2018; Zhou et al., 2021). With increasing forest age, the competitive relationships and resource utilization patterns within a stand change (Bongers et al., 2021). Plants in younger stands are usually more active, with potentially higher nutrient demands, resulting in relatively higher nitrogen content in their leaves (Miller, 1995; Carranca et al., 2018). As the forest ages, intensified competition among plants leads some to accumulate more nitrogen in their leaves to enhance their competitive ability, while others may reduce nitrogen content to conserve nutrients. Stand density is another indicator affecting leaf nutrient traits. Studies have shown that in high-density stands, photosynthesis and transpiration in leaves are significantly reduced, which in turn affects leaf nutrient traits. This implies that both the age and density of a forest can have profound impacts on the nutritional characteristics of its leaves, influencing overall forest health and productivity.

The differences in forest origin (planted forests vs. natural forests) also significantly impact leaf nutrient functional traits. One major distinction between planted and natural forests lies in their tree species composition and biodiversity. Planted forests are often characterized by a single or a limited number of tree species, whereas natural forests usually include a variety of different plant species, resulting in higher plant diversity (Kuuluvainen, 2009). This difference indirectly affects nutrient competition and distribution. In planted forests, the competition among the same tree species can be more intense, potentially leading some trees to accumulate more nitrogen and phosphorus in their leaves to enhance their competitive ability (Zhang et al., 2022). In natural forests, species diversity might lead to a more balanced resource allocation among different tree species, thereby affecting the stability of leaf nitrogen and phosphorus content and the N/P ratio (Augusto and Boča, 2022). Furthermore, soil properties and nutrient status also differ between planted and natural forests (Kooch et al., 2016). Planted forests often undergo soil improvement and fertilization as management measures to enhance tree growth rates (Fox, 2000). This could result in higher nitrogen and phosphorus content in the soil, thus increasing the nitrogen and phosphorus content in the leaves. In natural forests, soil nutrient conditions are typically influenced by natural processes and may exhibit significant variability (Thom and Seidl, 2016), which can also affect the stability of leaf nutrient functional traits.

**Figure 1:**
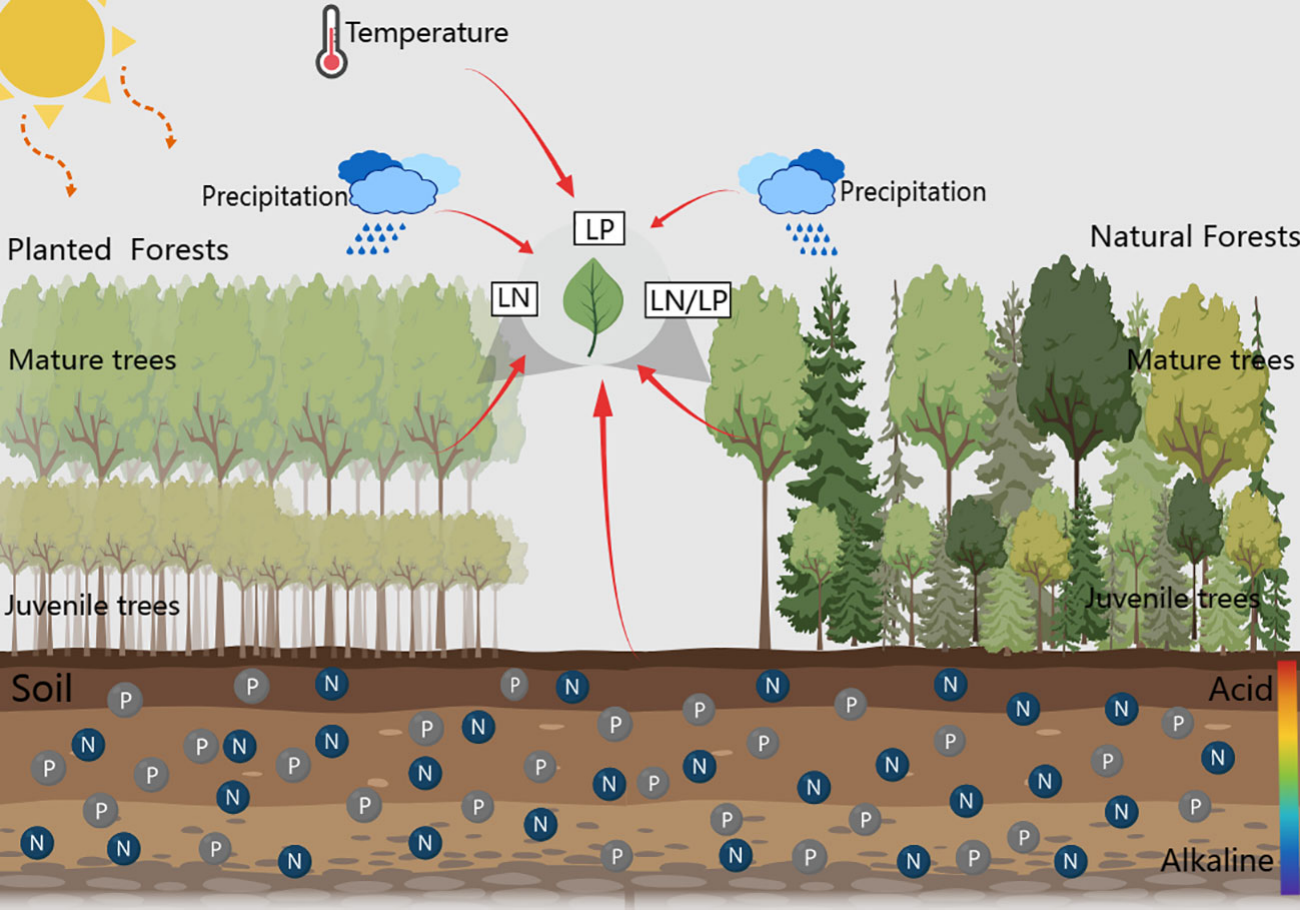
Graphical abstract of how climatic factors, soil nutrient factors, and stand factors influence the leaf nutrient traits of planted and natural forest.

**Figure 2:**
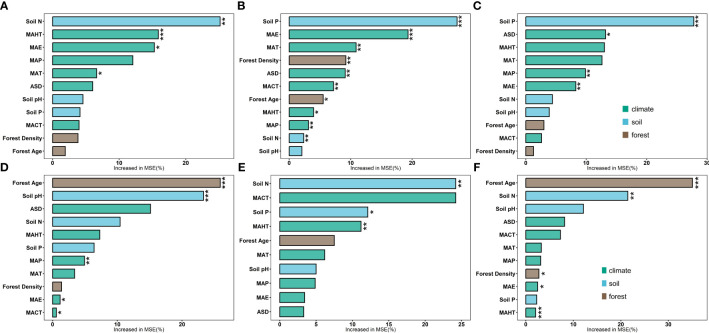
Boosted regression tree analysis model was employed to analyze the independent contributions of three categories of factors (climatic factors, soil factors, and stand factors) to the leaf nutrient traits of natural and planted forests. Brown represents stand factors, blue for soil factors, and green for climatic factors. LN in natural forests **(A)**, LP in natural forests **(B)**, leaf N/P ratio **(C)**, LN in planted forests **(D)**, LP in planted forests **(E)**, and leaf N/P ratio in planted forests **(F)** (***P< 0.001; **P< 0.01; *P< 0.05).

Based on field surveys and literature collected from 2005 to 2020 on 384 planted forests and 541 natural forests in China, this study aims to explore the spatial differences and dominant factors in leaf nutrient functional traits between planted and natural forests (**Figures 1**, **2E**). To address the above issues, we propose the following hypotheses: 1) There are significant differences in leaf nutrient traits between planted and natural forests at a macro scale. 2) Abiotic factors such as climatic factors and soil nutrients are the dominant factors controlling the large-scale spatial variability of leaf nutrient traits in planted and natural forests, with forest age also playing a significant role. 3) The direct effects of climatic factors on the spatial variability of leaf nutrient traits in planted and natural forests are greater than their indirect effects.

## Materials and methods

### Study area and sample data

**Figure 3:**
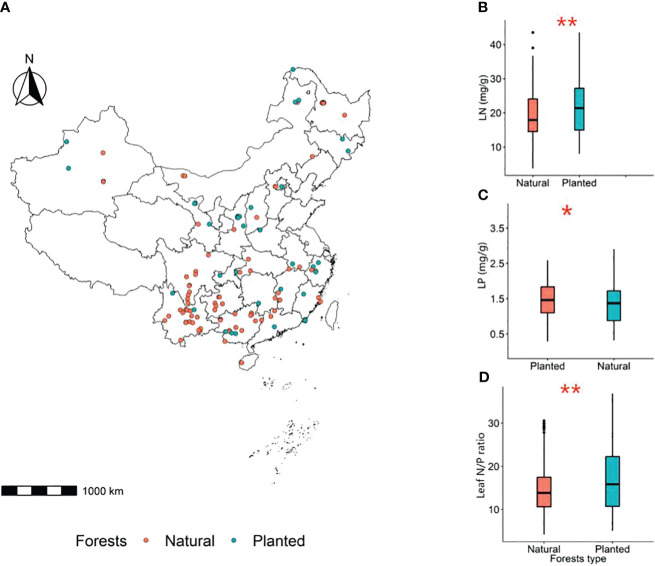
Spatial location of sampling sites in planted and natural forests in China **(A)**. Red circles represent natural forests and blue circles represent planted forests;. Comparison of LN **(B)**, LP **(C)** and leaf N/P ratio **(D)** between planted and natural forests, with the significance assessed at the 0.05 level. *P<0.05, **P<0.01.

China leads globally in planted forest acreage, a result of extensive afforestation programs (Guo and Ren, 2014; Gao et al., 2023). This study compares leaf nutrient traits between natural and planted forests, analyzing 386 planted and 540 natural forest sites established in China from 2005 to 2020 (**Figure 3A**). Data for the 926 forests were sourced from field surveys and literature, with detailed sources listed in **Supplementary Table S1**. 628 forest datasets from 73 sites were gathered through literature review, while the remaining 298 forest datasets from 21 sites were obtained from experiments conducted in this research. The collection and calculation methods for literature data are consistent with those of field experiment methods.

At each site, we randomly selected a minimum of four 30 m × 30 m plots, representative of the typical zonal vegetation. We recorded geographic coordinates, elevation, and plot slope. For each plot, we mapped the spatial distribution of trees, measuring diameter at breast height (DBH) and height for all trees ≥1 cm DBH. Species identification was done using scientific names, verified against authentic herbarium specimens.

### Key leaf nutrient traits

This study focuses on major leaf nutrient traits: leaf nitrogen content (LN) (g/kg), leaf phosphorus content (LP) (g/kg), and the leaf N/P ratio. The determination of leaf nitrogen content utilized the Kjeldahl method, a national standard procedure (Sáez-Plaza et al., 2013), while leaf phosphorus content was measured using the vanadium-molybdate colorimetric method (Hanson, 1950). The leaf N/P ratio is calculated as leaf nitrogen content divided by leaf phosphorus content.

Acknowledging the variation in tree species abundance, to mitigate the impact of asymmetric competition on community-level outcomes, the study employs Community Weighted Mean (CWM) traits to represent the average nutrient traits of a forest Equation 1.


(1) $$\text{CWM}=\sum_{\text{i}=1}^{\text{S}}\text{Di}\;\times \text{Traiti}$$

Where CWM denotes the community-weighted mean of nutrient traits (LN, LP, and the leaf N/P ratio), where D_i_ symbolizes the abundance of each tree species, and Trait_i_ refers to the specific leaf nutrient trait under consideration.

### Environmental data

We extracted the annual mean temperature (MAT), annual mean precipitation (MAP), maximum annual temperature (MAHT), and mean annual evapotranspiration (MAE) at a spatial resolution of 1 km from the WorldClim global climate layers. Annual sunshine duration (ASD) data was obtained from the China Meteorological Administration Climate Data Center (http://data.cma.cn/site/index.html). Soil pH, soil nitrogen, and available soil phosphorus were extracted from a 250-meter resolution grid, focusing on the top 30 cm of the soil layer. The data sources for soil nitrogen and available soil phosphorus are http://www.csdn.store and https://www.osgeo.cn/data/wc137, respectively.

### Data analysis

We employed t test at the 0.05 level to examine significant differences in LN, LP, and leaf N/P ratio between planted and natural forests. This analysis was conducted using the R package ‘agricolae’ (version 4.1.0).

We employed linear regression models to explore the impact of various environmental factors on leaf nitrogen (LN), leaf phosphorus (LP), and the leaf N/P ratio. These factors were categorized into climate factors, soil factors, and stand factors. Climate factors encompassed annual mean temperature (MAT), annual mean precipitation (MAP), mean temperature of the coldest month (MACT), and annual sunshine duration (ASD). Soil factors were represented by soil nitrogen content (Soil N), soil phosphorus content (Soil P), and soil pH. Stand factors considered included stand density and forest age. The goodness of fit for these models was assessed by the R² value, while their statistical significance was determined by the P value, using the ‘agricolae’ package (version 4.1.0) in R.

**Figure 4:**
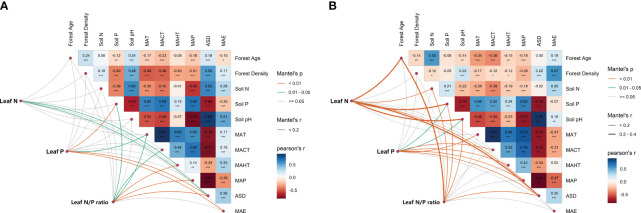
Multivariable correlation analysis of potential influencing factors of leaf nutrient traits (LN, LP and leaf N/P ratio) in natural **(A)** and planted **(B)** forests. The influencing factors include climate factors (MAT, MACT, MAHT, ASD, MAP and MAE), soil nutrient factors (Soil N, Soil P and Soil pH) and stand factors (Forest Age and Forest Density). LN in natural forests **(A)**, LN in planted forests **(B)**, LP in natural forests **(C)**, LP in planted forests **(D)**, leaf N/P ratio in natural forests **(E)**, and leaf N/P ratio in planted forests **(F)** (***P< 0.001; **P< 0.01; *P< 0.05).

To address the issue of multicollinearity, which arises from the presence of multiple dependent variables, we conducted Multivariate correlation analysis. This analysis aimed to elucidate the interrelationships among the dependent variables, as depicted in **Figure 4**. The ‘linkET’ package in R was utilized for this multivariate correlation analysis, ensuring a comprehensive examination of the variables’ interconnectedness.

Variance decomposition methods were used to quantify the explanatory power of various climate, soil, and stand factors on the spatial variability of key forest leaf nutrient traits. The variance decomposition analysis was conducted in the R language package ‘rdacca.hp’. The independent contribution of each potential influencing factor to the spatial variability of key leaf nutrient traits was explored using machine learning’s boosted regression tree analysis, applying a significance test at the 0.05 level. This analysis was completed using the R language package ‘gbm’.

Using segmented structural equation modeling (SEMs) to explore the pathways of influence of climatic factors, soil nutrient factors, and forest stand factors on key leaf nutrient traits. This approach allowed us to account for the random effects attributable to our sampling points and to quantitatively dissect the environmental factors into their ‘marginal’ and ‘conditional’ contributions. Our analytical process hinged on the utilization of the ‘piecewiseSEM,’ ‘nlme,’ and ‘lme4’ packages, each instrumental in refining our model’s accuracy and robustness. The adequacy and fit of our SEMs were critically evaluated using Fisher’s C-test, a statistical tool that helps determine the congruence between our data and the hypothesized model structure. We set stringent criteria for model acceptance, requiring a significance level of *P*< 0.05 and a satisfactory model goodness, indicated by a Fisher’s C/df ratio ranging from 0 to 2, and a *P*-value between 0.05 and 1.00. To optimize our model’s explanatory power and accuracy, we implemented a stepwise modification process, allowing for incremental adjustments based on these statistical benchmarks. This meticulous approach ensured that our final model reliably reflected the intricate interactions between ecological factors and key leaf nutrient traits.

## Results

The leaf nitrogen content (LN) and leaf N/P ratio of planted forests are significantly higher than those of natural forests, while the leaf phosphorus content (LP) is significantly lower than that of natural forests (*P*< 0.05) (**Figure 3**).

With increasing MAT and MAP, LN in planted forests significantly decreased (*P*< 0.001), whereas it significantly increased with longer sunlight duration (*P<* 0.001) (**Supplementary Figure S1**). In contrast, LN in natural forests did not show significant trends with climatic variations (*P* > 0.05) (**Supplementary Figure S1**), indicating greater climatic plasticity in LN of planted forests compared to natural forests. Leaf P content and leaf N/P ratio in both forest types exhibited opposite trends in response to climatic factors (**Supplementary Figures S2**, **S3**), with climate variables explaining a higher proportion of variation in natural forests (higher R^2^), suggesting greater climatic plasticity in these traits (**Supplementary Figures S1**–**S3**).

Soil nutrient content (nitrogen, phosphorus) and soil pH led to contrasting changes in key leaf nutrient traits between planted and natural forests. As the soil nitrogen and phosphorus content increases, the LN of planted forests significantly decreases, while the LN of natural forests does not show a significant trend of change (*P* > 0.05) (**Supplementary Figure S4**). Similarly, as the soil nitrogen and phosphorus content increases, the LP of natural forests does not show a significant trend of change either (*P* > 0.05). Overall, the effect of soil nitrogen and phosphorus content on the variability of LN in planted forests is greater than that on LP (with a higher R^2^).Soil nutrients had a greater impact on key leaf nutrient traits in planted forests than in natural forests (**Supplementary Figure S4**–**S6**).

Forest age had a stronger explanatory power for key leaf nutrient traits than stand density (**Supplementary Figure S7**–**S9**). With increasing forest age, leaf N and P contents significantly increased, and leaf N/P ratio significantly decreased in natural forests (*P<* 0.05) (**Supplementary Figures S7B**–**S9B**). Overall, Forest age of planted forests is generally lower than that of natural forests (**Supplementary Figure S9B**). The ability of stand factors (forest age and stand density) to shape the spatial variability of leaf nutrient traits in planted and natural forests is weaker than that of climatic and soil factors.

**Figure 5:**
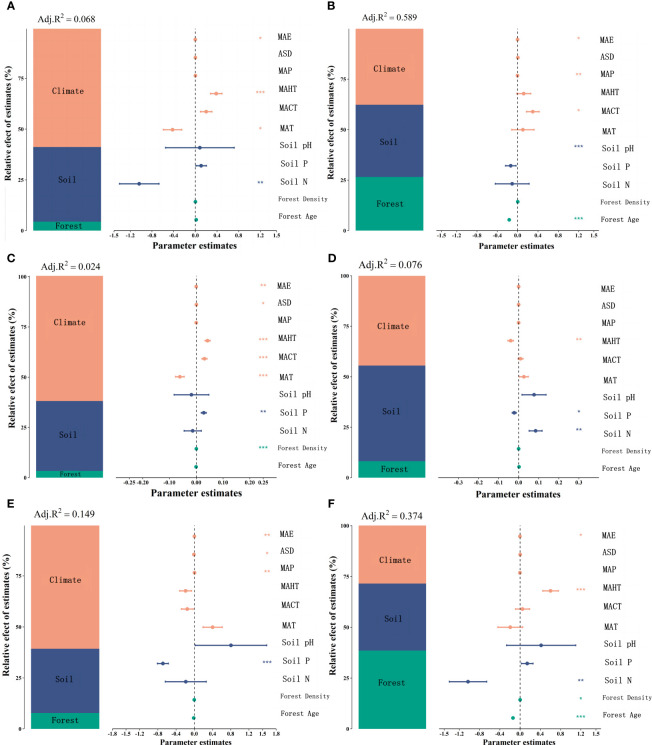
Variance decomposition analysis was used to explore the contributions of climatic factors, soil nutrient factors, and stand factors to leaf nutrient traits in natural and planted forests. The proportions of influence by each category of factors are shown, with orange representing climatic factors, blue for soil factors, and green for stand factors. Significance analysis is also presented, with asterisks indicating significance levels (***P< 0.001; **P< 0.01; *P< 0.05). LN in natural forests **(A)**, LN in planted forests **(B)**, LP in natural forests **(C)**, LP in planted forests **(D)**, leaf N/P ratio in natural forests **(E)**, and leaf N/P ratio in planted forests **(F)**.

**Figure 6:**
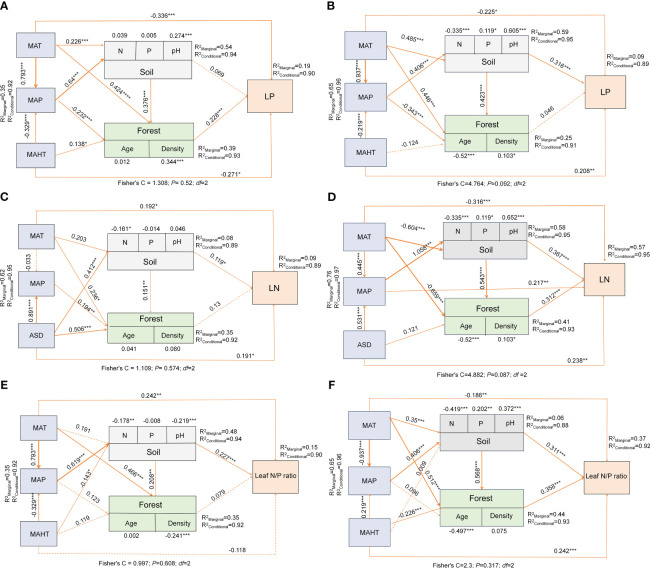
Linkages among climatic factors, soil nutrients, stand factors and leaf nutrient traits in China for natural and planted forests. Path diagrams represent the standardized results of final structural equation models (SEMs) examining the relationships among variables. Numbers alongside pathways indicate standardized SEM coefficients with asterisks indicating significance (***P< 0.001; **P< 0.01; *P< 0.05). R^2^ represents the generalized additive model (GAM) goodness-of-fit. The best SEMs was selected with the lowest AIC. LP in natural forest **(A)**, LP in planted forest **(B)**, LN in natural forest **(C)**, LN in planted forest **(D)**, Leaf N:P ratio in natural forest **(E)**, Leaf N:P ratio in planted forest **(F)**.

Significant correlations were observed among potential influencing factors of key leaf nutrient traits (**Figure 4**). Climate factors contributed more to key leaf nutrient traits than soil and stand factors (**Figure 5**). Overall, soil nutrient factors independently contributed the most to leaf nutrient traits in natural forests (**Figures 2A–C**), while stand factors had the largest independent contribution in planted forests (**Figures 2D, F**). Structural equation modeling results showed that climate factors not only directly affected key leaf nutrient traits in both planted and natural forests but also indirectly through soil and stand factors, with direct effects being more significant than indirect ones (**Figure 6**).

## Discussion

Leaves are the primary organs for photosynthesis in plants (Poorter et al., 2009), and leaf nutrient traits significantly influence forest productivity. Our study found that the nitrogen (N) and phosphorus (P) contents, as well as the N/P ratio in leaves of planted forests, are significantly higher than those in natural forests (**Figure 3**). This is mainly because planted forests, often located in urban or suburban areas, are more influenced by human activities and effective management, leading to a greater range of nutrient sources (Chen and Li, 2021; Abbasi et al., 2023). Furthermore, trees in planted forests are usually selected for their rapid growth, necessitating a higher intake of nutrients such as nitrogen and phosphorus for their biosynthetic processes. Typically characterized by monocultures or a limited variety of tree species (Liu et al., 2018), these species in planted forests may have genetic traits that enable higher nitrogen and phosphorus absorption and utilization efficiency. This results in significantly higher leaf nutrient traits in planted forests compared to natural forests.

With increasing precipitation and temperature, LN in the leaves of planted forests significantly decreases (**Supplementary Figure S1**). Increased precipitation enhances soil moisture availability, which may reduce plant nitrogen uptake and utilization efficiency (Wen et al., 2021). Planted forests exhibit higher climatic plasticity in leaf nitrogen content, while natural forests show greater climatic plasticity in leaf phosphorus content and leaf N/P ratio (Gao et al., 2023). Planted forests are often composed of artificially selected tree species, optimized to adapt to specific environmental conditions, including climate factors (Pawson et al., 2013). This selection likely enhances the adaptability and plasticity of planted forests to environmental changes, particularly in regulating nitrogen content. Planted forests receive more focused and purposeful management, such as regular fertilization, irrigation, and pest control (Hartley, 2002), which might affect plant nitrogen absorption and metabolism, thereby enhancing their ability to regulate nitrogen content under varying climatic conditions. Additionally, natural forests have higher species diversity and ecosystem complexity (Ehbrecht et al., 2021), leading to more variable and complex responses to climatic factors. This diversity may make natural forests more sensitive to climate changes in terms of leaf N/P ratio and phosphorus content.

Soil nutrients have a greater impact on key leaf nutrient traits in planted forests compared to natural forests (**Supplementary Figure S4**). This is mainly because plants in natural forests may possess more complex and diversified root structures, enabling them to absorb nutrients more effectively under nutrient-poor conditions. In contrast, tree species in planted forests might lack such strong root adaptability, making them more dependent on soil nutrients. Additionally, natural forest ecosystems typically have more complex nutrient cycling processes, including interactions among plants, microorganisms, and soil fauna. This complex nutrient cycling helps maintain nutrient balance within the ecosystem (Baldrian et al., 2023). Nutrient cycling in planted forests may be simpler (Ma et al., 2007), making them more sensitive to changes in external soil nutrients. Moreover, we found that with increasing forest age, nitrogen and phosphorus contents in leaves of natural forests significantly increase, while the leaf N/P ratio significantly decreases. As natural forests age, their ecosystems gradually mature, making internal nutrient cycling more efficient (Chen et al., 2020). Mature forests typically have deeper root systems and richer soil organic matter (Perry et al., 2012), which facilitate the accumulation and cycling of nutrients like nitrogen and phosphorus. Furthermore, ongoing organic matter decomposition and leaf litter accumulation during succession increase the organic matter content in the soil, enhancing soil fertility. More fertile soils can provide more nutrients such as nitrogen and phosphorus to plants (Furey and Tilman, 2021). The decrease in the leaf N/P ratio may be due to the rate of increase in soil phosphorus availability exceeding that of nitrogen as the forest ages. Additionally, plants in mature forests may require more phosphorus to support their complex physiological functions and maintain ecosystem stability.

At the macro scale, the explanatory power of climatic factors on leaf nutrient traits is stronger than that of soil nutrient factors and stand factors (**Figure 5**). This is mainly due to the extensive and profound influence of climatic factors (such as temperature, precipitation, and sunlight duration) on plant growth and metabolic processes at a global scale (Raza et al., 2019).Climatic conditions directly affect plant photosynthesis, respiration, transpiration, and nutrient uptake (Tkemaladze and Makhashvili, 2016), thereby influencing leaf nutrient traits. Additionally, climatic factors indirectly affect the physical and chemical properties of soil, including soil temperature, moisture, pH, and organic matter decomposition rate (Certini and Scalenghe, 2023), all of which impact the availability of soil nutrients. Therefore, climatic factors to some extent determine the status of soil nutrients. Compared to climatic factors, soil and stand factors may have more limitations and heterogeneity at the spatial scale. Soil characteristics are influenced by geographic location, topography, and parent rock type, while stand factors (such as forest age, density, and species composition) vary greatly across different regions and forest types.

Climatic factors not only have a direct effect on leaf nutrient traits but also indirectly influence them through impacts on soil nutrients and stand factors, with their direct effects being more pronounced than the indirect ones (**Figure 6**). This is primarily because the regulation and metabolism of leaf nutrients in plants are direct physiological responses to current climatic conditions (Wang et al., 2022), characterized by rapid response and strong regulatory capacity. Additionally, the impact of climate on soil nutrients and stand structure has a certain time lag (Spohn et al., 2023). Changes in soil characteristics and stand structure usually take a longer time; therefore, their impact on leaf nutrient traits is more indirect and gradual. Global trends and patterns in climatic factors comprehensively affect the growth environment of plants, while changes in soil and stand factors are often more localized and specific. Consequently, at a macro scale, the direct influence of climatic factors on the key leaf nutrient traits of planted and natural forests is typically greater than their indirect impact through soil nutrient and stand factors. This direct effect reflects the plants’ ability to rapidly and directly respond to current and specific climatic conditions.

## Data availability statement

The raw data supporting the conclusions of this article will be made available by the authors, without undue reservation.

## Author contributions

XZ: Writing – review & editing, Writing – original draft, Supervision, Project administration. MY: Writing – review & editing, Writing – original draft, Investigation, Conceptualization. JXS: Writing – review & editing, Writing – original draft, Methodology, Formal analysis, Conceptualization. JX: Writing – review & editing, Writing – original draft, Supervision, Project administration. XTZ: Writing – review & editing, Writing – original draft, Validation, Formal analysis. JLS: Writing – review & editing, Writing – original draft, Software, Investigation, Conceptualization. JG: Writing – review & editing, Writing – original draft, Supervision, Methodology, Data curation.

## Supplementary material

Supplementary Figure 1 Linear relationships between LN and MAP **(A)**, MAT **(B)**, MACT **(C)** and ASD **(D)**. Red indicates natural forests, while blue represents planted forests. R^2^ denotes the model’s fit, and *P* represents the correlation significance.

Supplementary Figure 2 Linear relationships between LP and MAP **(A)**, MAT **(B)**, MACT **(C)** and ASD **(D)**. Red indicates natural forests, while blue represents planted forests. R^2^ denotes the model’s fit, and *P* represents the correlation significance.

Supplementary Figure 3 Linear relationships between Leaf N/P ratio and MAP **(A)**, MAT **(B)**, MACT **(C)** and ASD **(D)**. Red indicates natural forests, while blue represents planted forests. R^2^ denotes the model’s fit, and *P* represents the correlation significance.

Supplementary Figure 4 Linear relationships between LN and soil N content **(A)** , soil P content **(B)** and soil pH **(C)**. Red indicates natural forests, while blue represents planted forests. R^2^ denotes the model’s fit, and *P* represents the correlation significance.

Supplementary Figure 5 Linear relationships between LP and soil N content **(A)**, soil P content **(B)** and soil pH **(C)**. Red indicates natural forests, while blue represents planted forests. R^2^ denotes the model’s fit, and *P* represents the correlation significance.

Supplementary Figure 6 Linear relationships between leaf N/P ratio and soil N content **(A)**, soil P content **(B)** and soil pH **(C)**. Red indicates natural forests, while blue represents planted forests. R^2^ denotes the model’s fit, and *P* represents the correlation significance.

Supplementary Figure 7 Linear relationships between LN and forest age **(A)** and forest density **(B)**. Red indicates natural forests, while blue represents planted forests. R^2^ denotes the model’s fit, and *P* represents the correlation significance.

Supplementary Figure 8 Linear relationships between LP and forest age **(A)** and forest density **(B)**. Red indicates natural forests, while blue represents planted forests. R^2^ denotes the model’s fit, and *P* represents the correlation significance.

Supplementary Figure 9 Linear relationships between leaf N/P ratio and forest age **(A)** and forest density **(B)**. Red indicates natural forests, while blue represents planted forests. R^2^ denotes the model’s fit, and *P* represents the correlation significance.
