# Supplementary figures and images for: Leaf nutrient traits of planted forests demonstrate a heightened sensitivity to environmental changes compared to natural forests
Source: Front Plant Sci. 2024 Mar 18;15:1372530. doi: 10.3389/fpls.2024.1372530 (PMC10982418; doi:10.3389/fpls.2024.1372530)

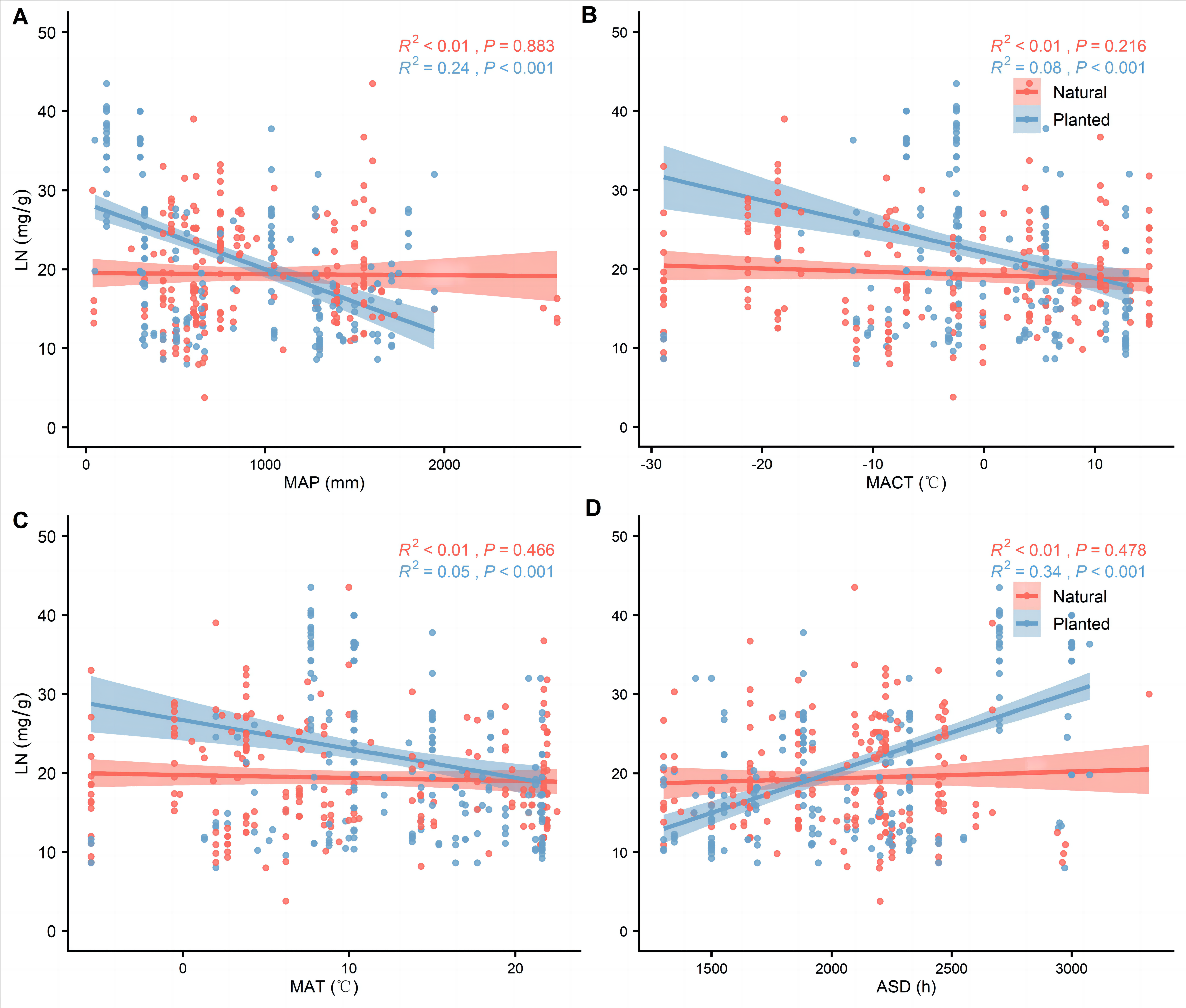

Supplement: Supplementary Figure 1 — Linear relationships between LN and MAP (A), MAT (B), MACT (C) and ASD (D). Red indicates natural forests, while blue represents planted forests. R2 denotes the model’s fit, and P represents the correlation significance. [file Presentation_1.zip › ╕▒═╝/Fig.S1.png]

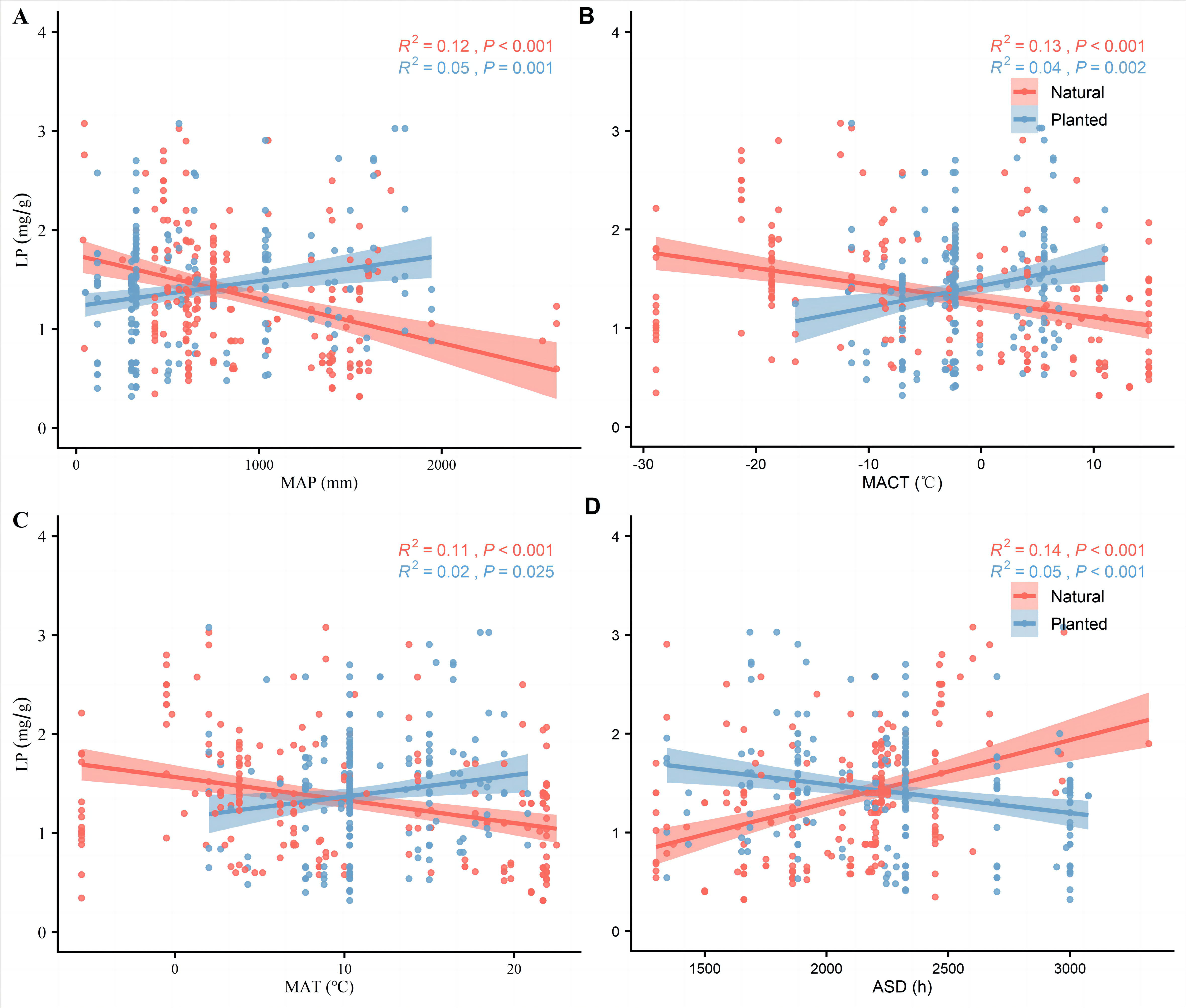

Supplement: Supplementary Figure 1 — Linear relationships between LN and MAP (A), MAT (B), MACT (C) and ASD (D). Red indicates natural forests, while blue represents planted forests. R2 denotes the model’s fit, and P represents the correlation significance. [file Presentation_1.zip › ╕▒═╝/Fig.S2.png]

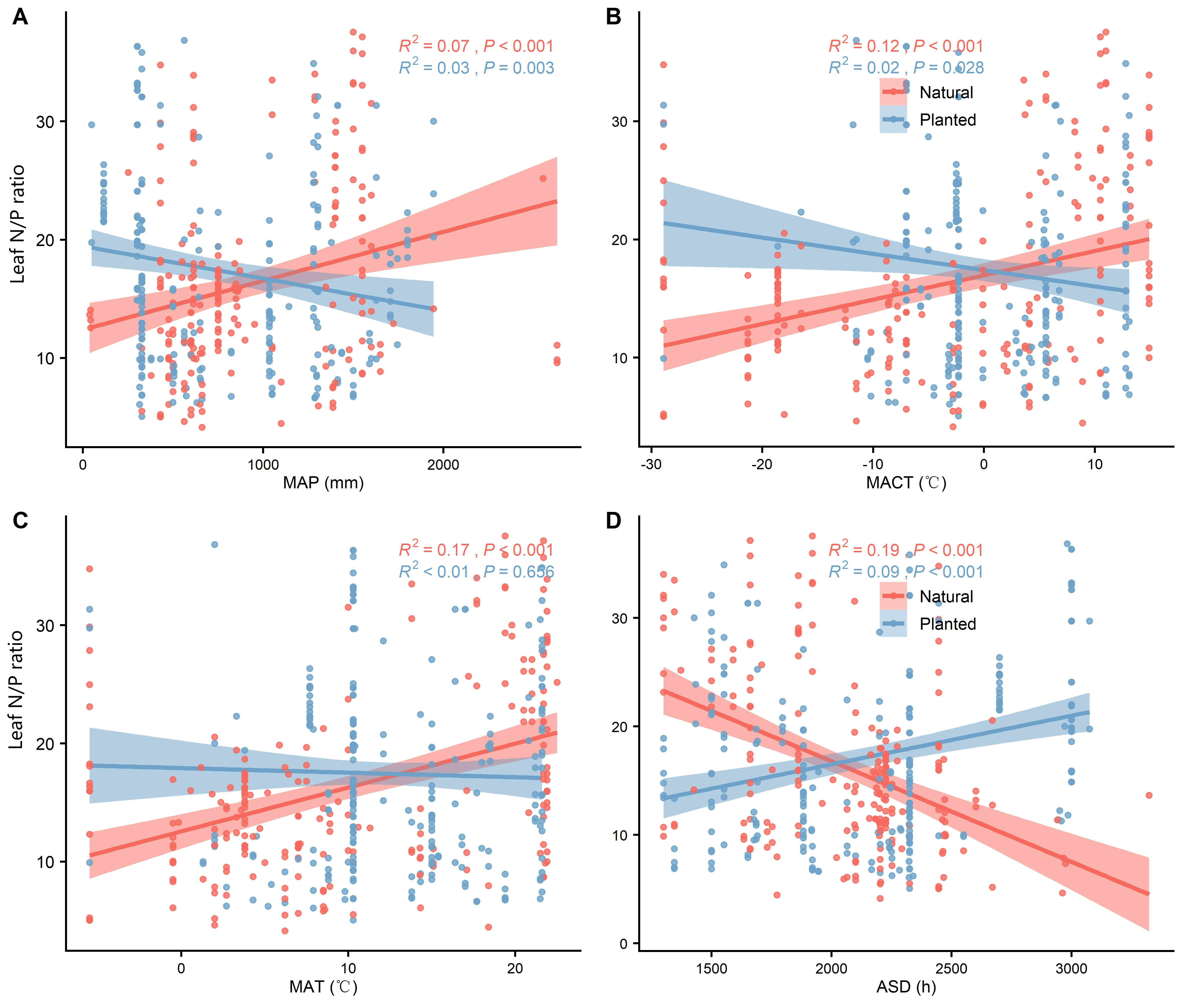

Supplement: Supplementary Figure 1 — Linear relationships between LN and MAP (A), MAT (B), MACT (C) and ASD (D). Red indicates natural forests, while blue represents planted forests. R2 denotes the model’s fit, and P represents the correlation significance. [file Presentation_1.zip › ╕▒═╝/Fig.S3.png]

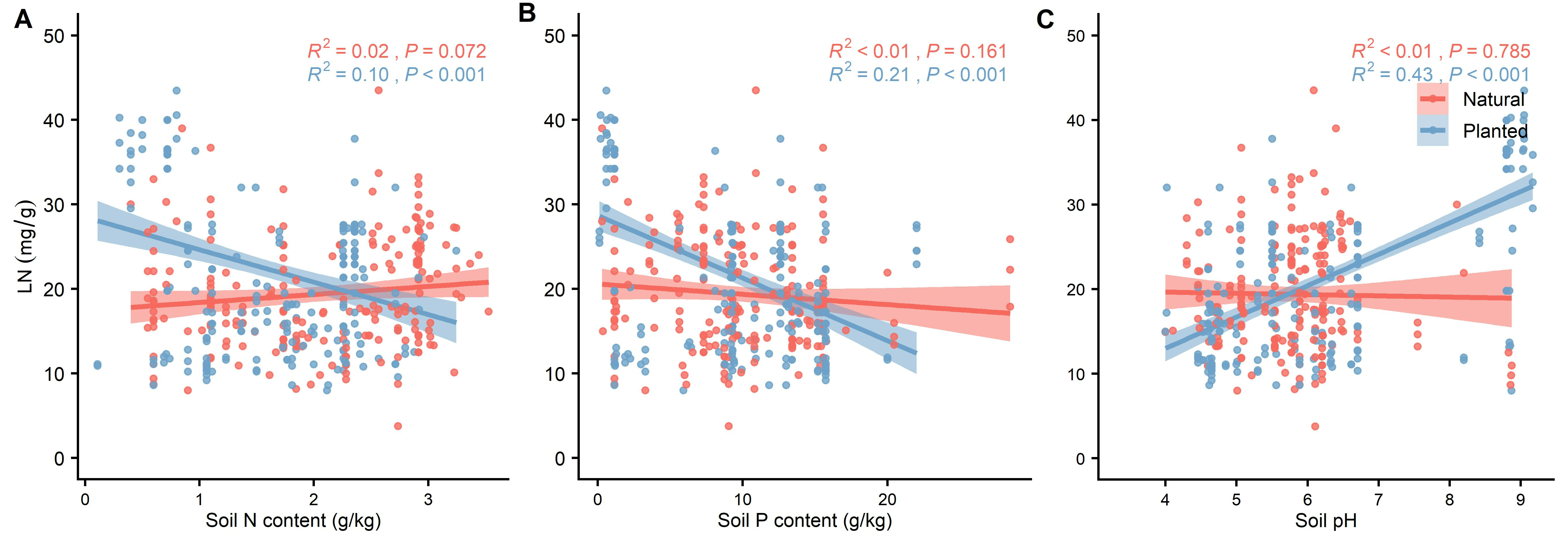

Supplement: Supplementary Figure 1 — Linear relationships between LN and MAP (A), MAT (B), MACT (C) and ASD (D). Red indicates natural forests, while blue represents planted forests. R2 denotes the model’s fit, and P represents the correlation significance. [file Presentation_1.zip › ╕▒═╝/Fig.S4.png]

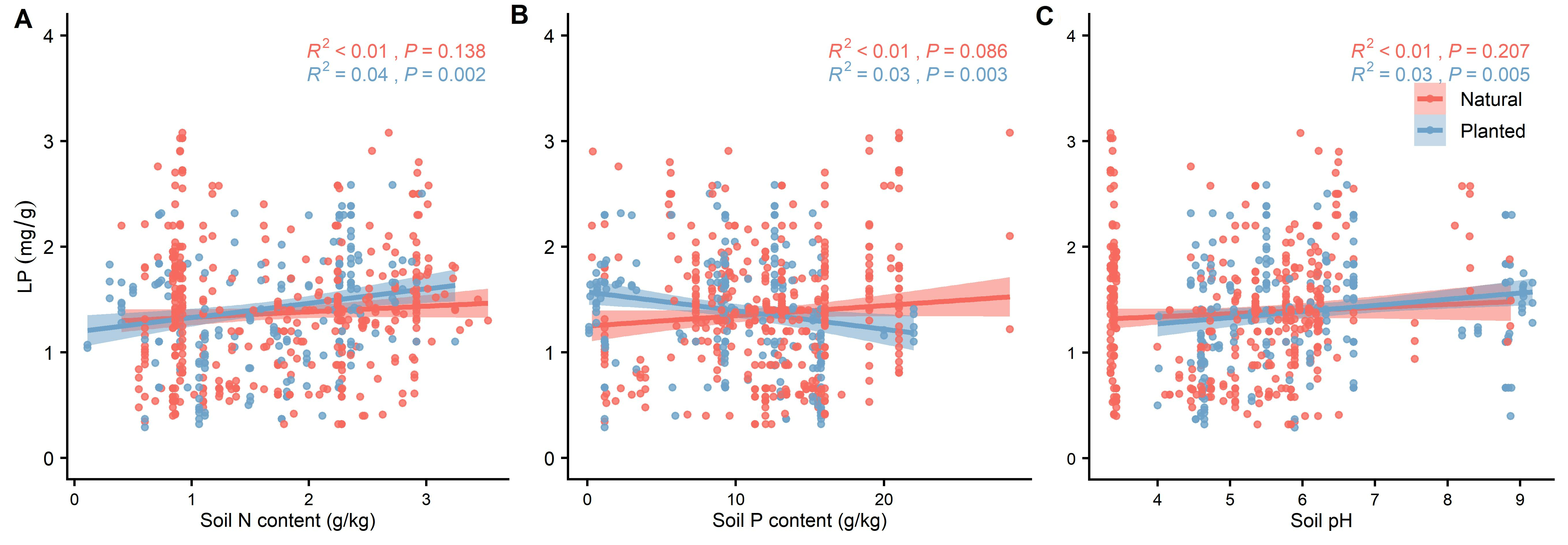

Supplement: Supplementary Figure 1 — Linear relationships between LN and MAP (A), MAT (B), MACT (C) and ASD (D). Red indicates natural forests, while blue represents planted forests. R2 denotes the model’s fit, and P represents the correlation significance. [file Presentation_1.zip › ╕▒═╝/Fig.S5.png]

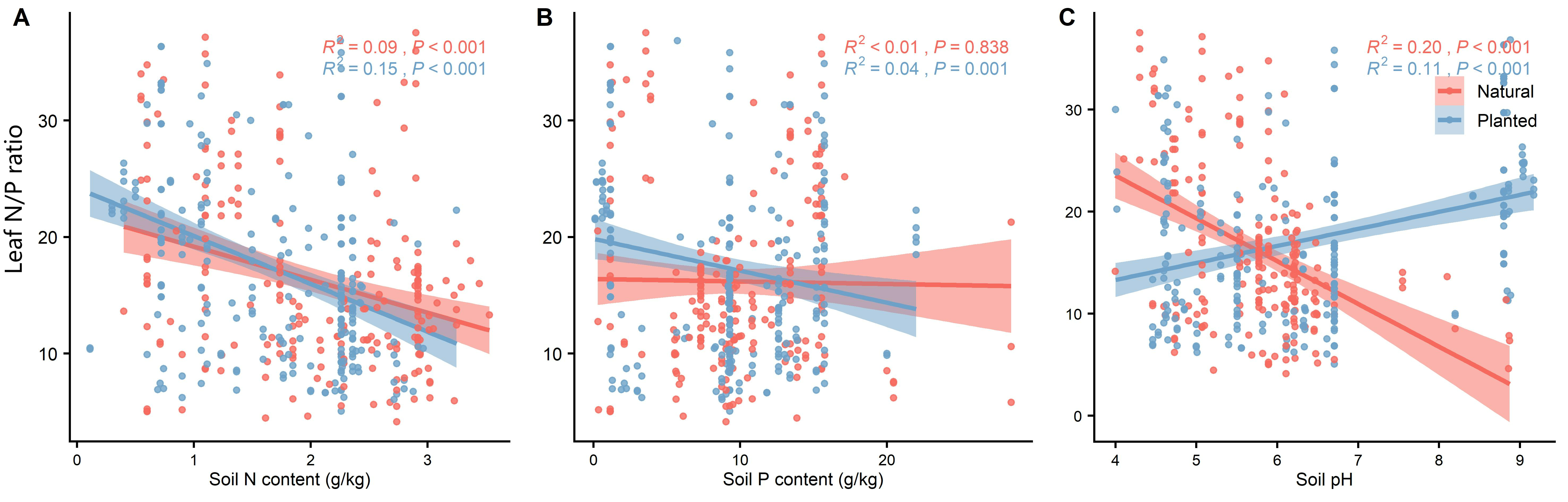

Supplement: Supplementary Figure 1 — Linear relationships between LN and MAP (A), MAT (B), MACT (C) and ASD (D). Red indicates natural forests, while blue represents planted forests. R2 denotes the model’s fit, and P represents the correlation significance. [file Presentation_1.zip › ╕▒═╝/Fig.S6.png]

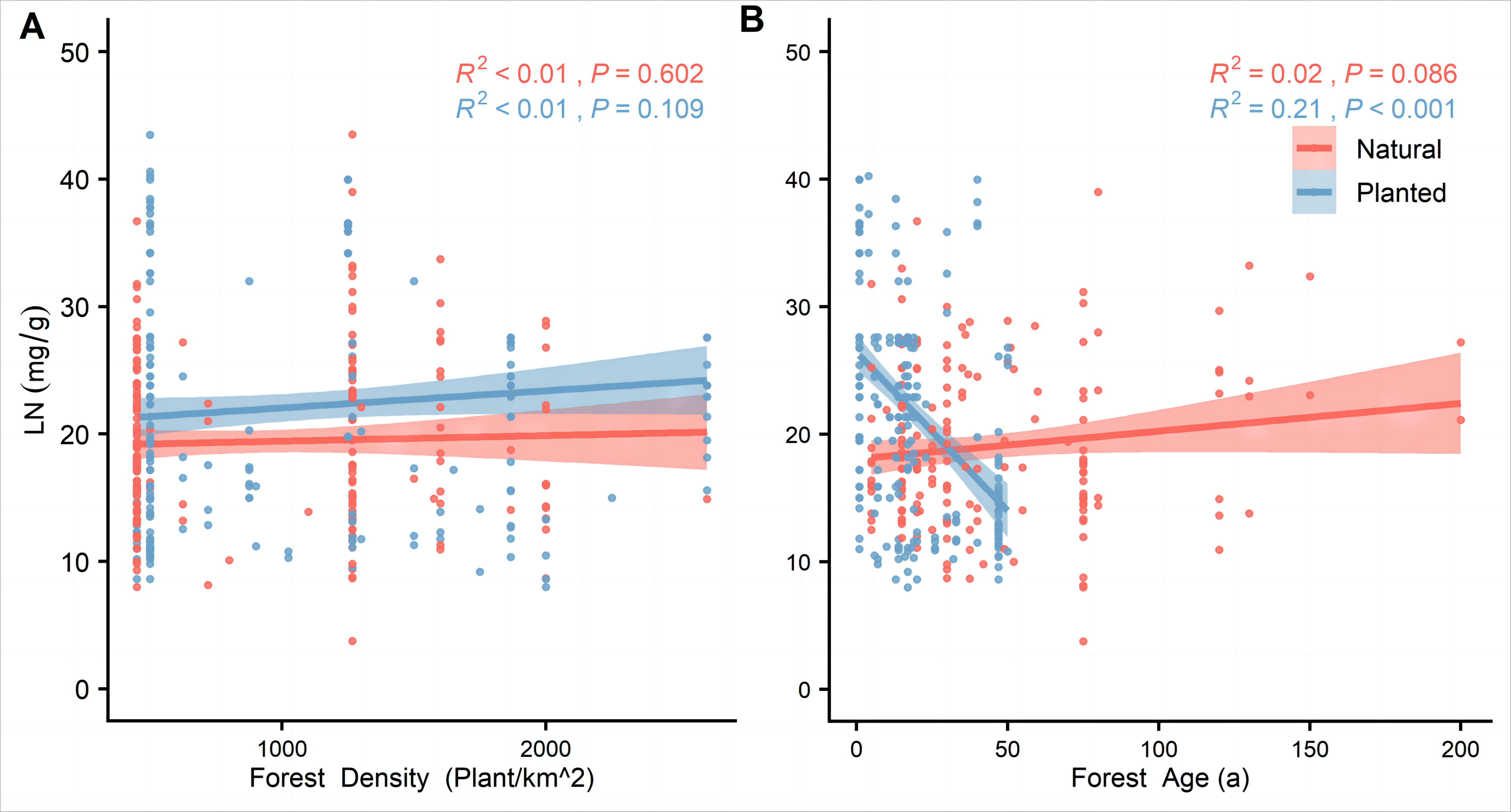

Supplement: Supplementary Figure 1 — Linear relationships between LN and MAP (A), MAT (B), MACT (C) and ASD (D). Red indicates natural forests, while blue represents planted forests. R2 denotes the model’s fit, and P represents the correlation significance. [file Presentation_1.zip › ╕▒═╝/Fig.S7.png]

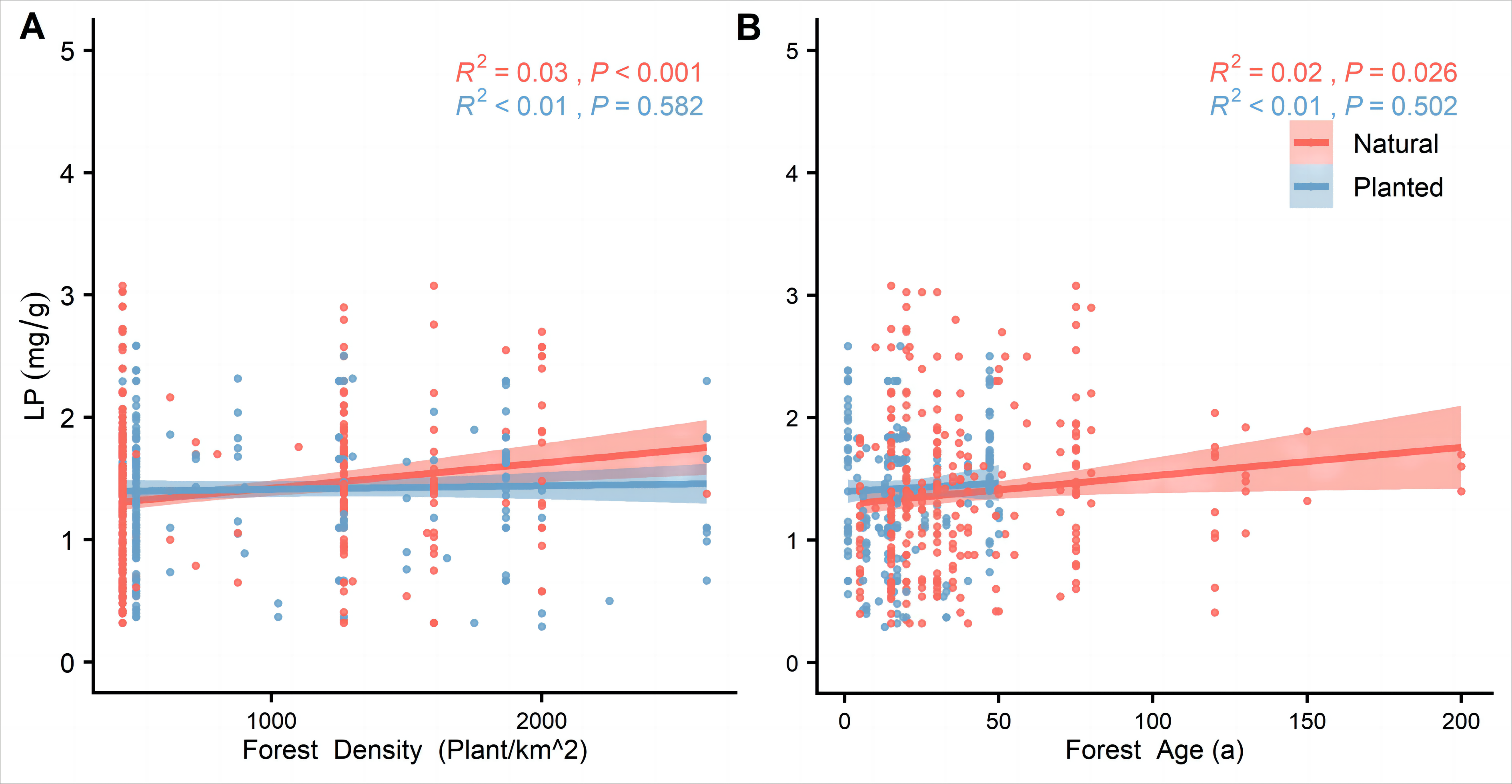

Supplement: Supplementary Figure 1 — Linear relationships between LN and MAP (A), MAT (B), MACT (C) and ASD (D). Red indicates natural forests, while blue represents planted forests. R2 denotes the model’s fit, and P represents the correlation significance. [file Presentation_1.zip › ╕▒═╝/Fig.S8.png]

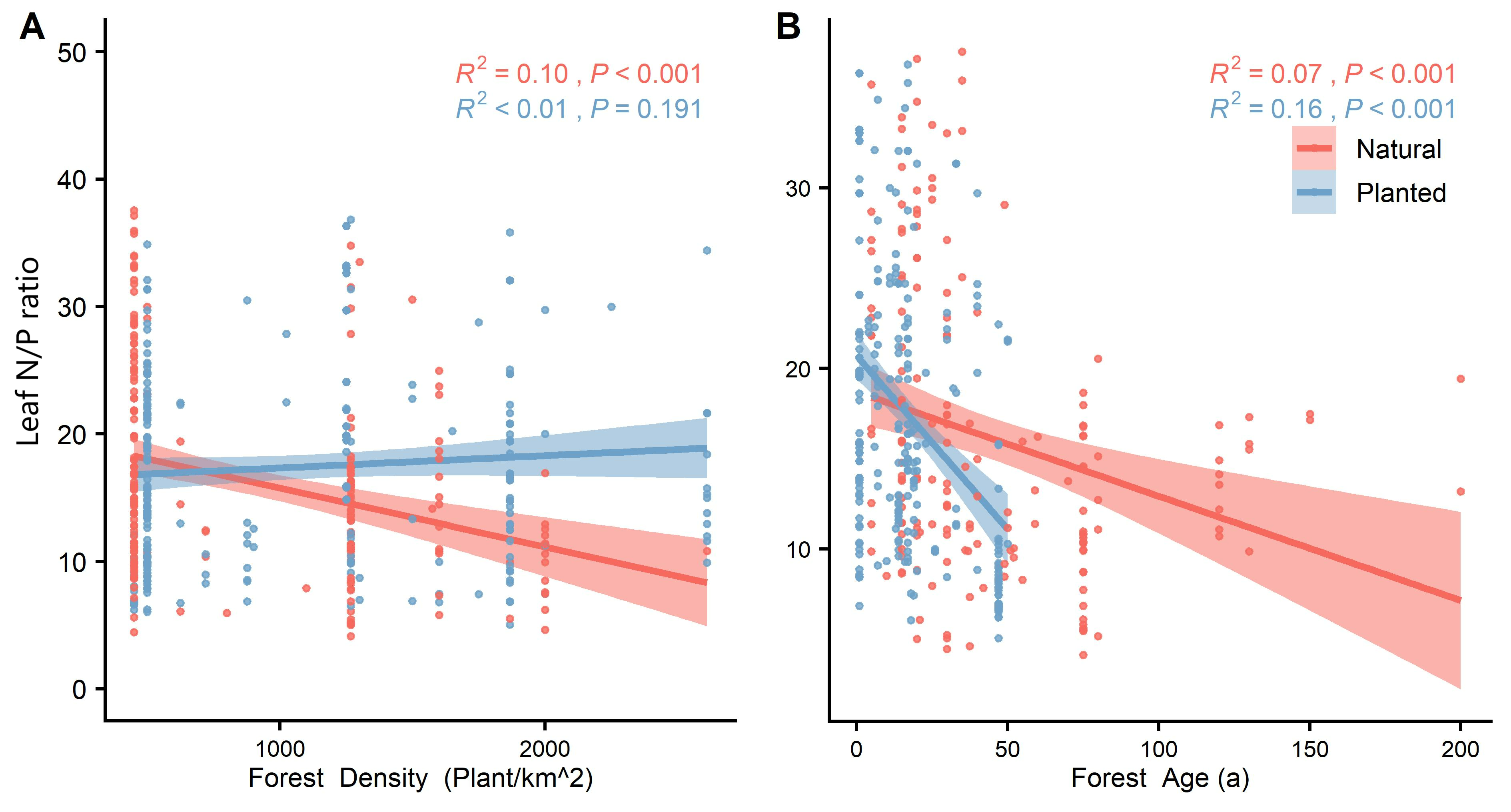

Supplement: Supplementary Figure 1 — Linear relationships between LN and MAP (A), MAT (B), MACT (C) and ASD (D). Red indicates natural forests, while blue represents planted forests. R2 denotes the model’s fit, and P represents the correlation significance. [file Presentation_1.zip › ╕▒═╝/Fig.S9.png]
